# Altered intrinsic and network properties of neocortical neurons in the Ts65Dn mouse model of Down syndrome

**DOI:** 10.14814/phy2.12655

**Published:** 2015-12-23

**Authors:** Nathan P. Cramer, Xiufen Xu, Tarik F. Haydar, Zygmunt Galdzicki

**Affiliations:** ^1^ Department of Anatomy, Physiology, and GeneticsF. Edward Hébert School of Medicine and Center for Neuroscience and Regenerative MedicineUniformed Services University of the Health SciencesBethesdaMarylandUSA; ^2^ Department of Anatomy and NeurobiologyBoston University School of MedicineBostonMassachusettsUSA

**Keywords:** Down syndrome, electrophysiology, neocortex, network, sensory, trisomy, Ts65Dn, Up states

## Abstract

All individuals with Down syndrome (DS) have a varying but significant degree of cognitive disability. Although hippocampal deficits clearly play an important role, behavioral studies also suggest that deficits within the neocortex contribute to somatosensory deficits and impaired cognition in DS. Using thalamocortical slices from the Ts65Dn mouse model of DS, we investigated the intrinsic and network properties of regular spiking neurons within layer 4 of the somatosensory cortex. In these neurons, the membrane capacitance was increased and specific membrane resistance decreased in slices from Ts65Dn mice. Examination of combined active and passive membrane properties suggests that trisomic layer 4 neurons are less excitable than those from euploid mice. The frequencies of excitatory and inhibitory spontaneous synaptic activities were also reduced in Ts65Dn neurons. With respect to network activity, spontaneous network oscillations (Up states) were shorter and less numerous in the neocortex from Ts65Dn mice when compared to euploid. Up states evoked by electrical stimulation of the ventrobasal nucleus (VBN) of the thalamus were similarly affected in Ts65Dn mice. Additionally, monosynaptic EPSCs and polysynaptic IPSCs evoked by VBN stimulation were significantly delayed in layer 4 regular spiking neurons from Ts65Dn mice. These results indicate that, in the Ts65Dn model of DS, the overall electrophysiological properties of neocortical neurons are altered leading to aberrant network activity within the neocortex. Similar changes in DS individuals may contribute to sensory and cognitive dysfunction and therefore may implicate new targets for cognitive therapies in this developmental disorder.

## Introduction

Down syndrome (DS), which results from triplication of genes on human chromosome 21 (Hsa21) is associated with significant cognitive deficits mainly in spatial cognition mediated by the hippocampus (Chapman and Hesketh 2000; Pennington et al. 2003; Edgin et al. 2010; Haydar and Reeves 2012). Morphological and functional abnormalities throughout the nervous system but particularly within the hippocampus have been implicated as contributing factors to diminished cognitive capabilities in DS individuals (Sylvester 1983; Ferrer and Gullotta 1990; Pinter et al. 2001; Smigielska‐Kuzia et al. 2011). Investigations using mouse models of DS indicate that hippocampal networks are indeed functionally altered with a diminished capacity for synaptic plasticity and alterations in plasticity‐related signaling pathways (Siarey et al. 1997, 2005, 2006; Kleschevnikov et al. 2004; Costa and Grybko 2005; Belichenko et al. 2009a; Cramer and Galdzicki 2012).

The neocortex also contributes significantly to cognition at many levels including working memory (Miller et al. 1996; Sawaguchi and Yamane 1999) and consolidation of short‐term into long‐term memories (Takashima et al. 2009; Nadel et al. 2012). Indeed, cognitive deficits in verbal and spatial short/long‐term memory have been reported in DS individuals with a severity that correlates with gray matter densities in neocortical regions (Menghini et al. 2011). Deficits in processing and responding to somatosensory information are also prevalent in individuals with Down syndrome (Bruni et al. 2010; Wuang and Su 2011). As it has been postulated that working memory may rely not just on the prefrontal cortex but on modality‐specific regions (Postle 2006; Bancroft et al. 2014) these deficits involving multiple areas of the DS neocortex would be expected to have adverse effects on both perception and memory formation/retention.

Delays in sensory‐evoked potentials recorded over the somatosensory cortex in DS infants (Chen and Fang 2005) suggest that changes in the electrophysiological nature of cortical networks are altered by aneuploidy of chromosome 21. These alterations may arise from fine structural changes at the subcellular level with altered cortical dendritic spine morphology and structural cortical dysgenesis present in the DS brain early in development (Marin‐Padilla 1972, 1976; Golden and Hyman 1994). As in the hippocampus where a proper balance between excitation and inhibition is important for effective network functioning (Siarey et al. 1997; Belichenko et al. 2009b; Chakrabarti et al. 2010; Best et al. 2012) cognitive processes within the neocortex also likely depend upon a balanced ratio of these opposing drives (Haider et al. 2006). Morphological deficits in DS individuals (Purpura 1975; Sylvester 1983; Becker et al. 1991) and mouse models (Belichenko et al. 2007, 2009b) suggest that the balance between excitation and inhibition may be altered by trisomy, an effect that could significantly contribute to cognitive deficits. However, unlike the hippocampus, our understanding of the functional impact of DS aneuploidy on neocortical networks is comparatively limited.

To address this knowledge gap of how information flow though the neocortex is altered by DS, we examined spontaneous and evoked network activity in thalamocortical slices from Ts65Dn mice, currently the most widely investigated mouse model of DS. Under conditions that closely mimic the natural cerebrospinal fluid environment, neocortical slices produce periodic oscillations of increased network activity (Up states) followed by periods of relative quiescence (Down states) (Sanchez‐Vives and McCormick 2000). The mechanisms underlying the generation of these oscillations are believed to reflect those used in cognitive processes such as working memory and memory consolidation (Haider et al. 2006) and are common to many regions of the neocortex (Ruiz‐Mejias et al. 2011) suggesting their pivotal role in a spectrum of cognitive processes. We find that baseline synaptic activity in layer 4 cells of the somatosensory/barrel cortex is reduced in slices from trisomic mice and that the intrinsic properties of layer 4 regular spiking neurons are perturbed in a manner that suggests they have a reduced capacity for sustained activity. We also observed changes in the kinetics of spontaneously occurring or stimulation evoked Up/Down states when compared to euploid littermates. These results suggest that overexpression of trisomic genes significantly alters the functional properties of neocortical networks and perturbs their development. These changes likely directly impact and/or exacerbate behavioral deficits associated with aberrant hippocampal networks and therefore implicate abnormal cortical network activity as an important contributor to DS cognitive deficits.

## Materials and Methods

### Animals

Ts65Dn (Jackson Laboratories, Bar Harbor ME) and control diploid littermates were bred to have the mixed genetic background C57BL/6JEi×C3H/HeSnJ as used in our previous studies (Siarey et al. 1997; Harashima et al. 2006b; Best et al. 2012). PCR genotyping was performed on genomic DNA extracted from tail tips according to the methods described in Lorenzi et al. (2010) or Reinholdt et al. (2011). Mice were maintained under a 12‐h light/dark cycle and fed standard laboratory food (following NIH guidelines). All protocols were approved by the Uniformed Services University of the Health Sciences Institutional Animal Care and Use Committee.

### Electrophysiology

Mice, 13 to 21 days old, were overdosed on isoflurane before transcardial perfusion with ice‐cold sucrose artificial cerebrospinal fluid (sACSF) containing (in mmol/L) sucrose 206, KCl 2, CaCl_2_ 1, NaH_2_PO_4_ 1.25, MgSO_4_ 2, MgCl‐6H_2_O 2, NaHCO3 26, D‐glucose 10, and bubbled with a mixture of 95% O_2_/5% CO_2_. The mice were then decapitated and the brains placed in a dish of sACSF on ice for blocking. Thalamocortical slices (Agmon and Connors 1991), 400‐*μ*m thick, were cut on a Leica VT1200S and transferred to a warmed (~36°C) solution of normal ACSF (nACSF) NaCl 126, KCl 3, CaCl_2_ 2, NaH_2_PO_4_ 1.25, MgSO_4_ 2, NaHCO_3_ 26, d‐glucose 10, bubbled with a mixture of 95% O_2_/5% CO_2_ for 45 min. After this recovery period the slices were maintained in the same solution at room temperature for at least 1 h before recording.

For recording, slices were placed in a chamber under an upright Zeiss FS‐1 microscope (Carl Zeiss Microimaging Inc., Thornwood, NY) and continuously perfused with ACSF consisting of (mmol/L): NaCl 124, KCl 4, CaCl_2_ 1, NaH_2_PO_4_ 1.25, MgSO_4_ 1, NaHCO_3_ 26, D‐glucose 10, bubbled with a mixture of 95% O_2_/5% CO_2_ (Sanchez‐Vives and McCormick 2000). Using a Photonics IR camera, layer 4 neurons of the somatosensory cortex were identified and a whole‐cell patch‐clamp configuration was obtained with a borosilicate patch pipette of resistance 3–5 MΩ containing (in mmol/L): K‐gluconate 130, KCl 15, HEPES 5, EGTA 1, Mg‐ATP 4, Na‐GTP 0.3 with pH adjusted to ~7.3 with KOH. A subset of recordings were obtained with an intracellular solution containing (in mmol/L) Cs‐gluconate 130, CsCl 2, NaCl 2, HEPES 10, EGTA 0.2, Mg‐ATP 4, Na‐GTP 0.3, QX‐314‐Cl 2 with pH adjusted to ~7.3 with CsOH. This allowed us to carry‐out recordings in voltage‐clamp configuration from the neurons at the empirically determined reversal potential for inhibitory (−45 mV) and excitatory components (20 mV) in order to isolate each component and assess their impact on overall electrical activity. Field potential recordings were obtained with a low impedance borosilicate pipette (~ 1MΩ) filled with the ACSF used for recording. Recordings were acquired by way of an Axopatch 200A, 200B, or 700B amplifier (Molecular Devices, Sunnyvale, CA), filtered at 5 kHz (8‐pole Bessel filter, NPI, ALA Scientific Instruments, Inc., Westbury, NY), and recorded on a personal computer using pClamp module of Clampex software (Molecular Devices).

To evoke Up states, a custom made bipolar tungsten electrode was placed in the ventrobasal (VB) nucleus of the thalamus. Stimuli (4 monophasic pulses, 200 *μ*s in duration at 40 Hz) were applied using a constant current stimulus isolator controlled by pClamp software. Stimulation intensities ranged from 10 to 800 *μ*A.

### Data analysis

Up states were identified using custom written functions in Igor (Wavemetrics, Portland OR). For field potential recordings, data were imported into Igor using the interface software DataAccess (Bruxton Corporation, Seattle WA), band pass filtered between 3 and 300 Hz and rectified. A period of network inactivity was visually identified and the standard deviation of the waveform from this region used as a baseline to scan the remaining data for Up states. Each signal was examined in 10 ms increments and, if the standard deviation exceeded 5‐times the baseline value for a minimum of 300 ms the electrical activity was marked as Up state. A transition back to a Down state was taken as a return of the signal below the threshold value for more than 100 ms. Up states that occurred within 1 sec of each other were considered to belong to the same event. Intracellular recordings were similarly analyzed for Up states except that the data was not band pass filtered or rectified. Transmission latencies were measured intracellularly in layer 4 cells with consistent short latency response produced by stimulation of VB. Layer 4 neurons were selected within visually identified barrels of the somatosensory cortex and the depth from the pia and cell density was used to target layer 4. A response was classified as monosynaptic if the coefficient of variation (CV) of the excitatory postsynaptic potential latency was less than 10%. Since, in barrel cortex, inhibitory synaptic inputs are accepted to be polysynaptic (Agmon and Connors 1991; Agmon et al. 1996), the CV restriction was not applied to these evoked responses. The onset of the response was taken as the point where the signal exceeded the preceding 5 ms of data by 3 standard deviations. Spontaneous synaptic activity was analyzed by isolating individual synaptic events in MiniAnalysis (SynaptoSoft, Decatur GA). This software analyzes continuously acquired membrane potential/current recordings and isolates individual synaptic events based on multiple shape criteria. For each synaptic event, the time of occurrence, amplitude and rise/decay times are recorded. Analysis of the frequency of synaptic events is based on the timing information acquired in this manner.

Intrinsic properties were analyzed with functions written in Igor. Only regular spiking neurons with resting potentials more negative than −55 mV were analyzed. These neurons were presumed to be excitatory neurons based on their spiking pattern. Fast spiking cells, were defined as neurons with firing frequencies above 60 Hz and with afterhyperpolarizations less than −40 mV, when evoked by stimuli 2‐fold of rheobase. Neurons that fired a spike doublet at the start of a depolarizing step were excluded from analysis. Rheobase was measured as the minimum current of 250 ms duration to evoke action potential from a holding potential of −70 mV. The threshold for action potential initiation was determined from the plot of dV versus V. The amplitude of the action potential was measured from this threshold value to the peak of the waveform. The full width at half max (FWHM) of the action potential was measured halfway between the threshold and the peak. After hyperpolarization amplitudes were measured from the action potential threshold to the minimum of the first trough following the spike. Spike frequency adaptation ratios were calculated as the ratio between the last two inter‐spike intervals divided by the first two interspike intervals at the rheobase current for each cell. Cell membrane resistance and capacitance were measured using the membrane‐test protocol in Clampex module. The membrane potential was measured in current‐clamp configuration as zero current potential (*I* = 0 mode). Comparisons between trisomic and euploid mice were made with Student's *t*‐test and Kruskal–Wallis one‐way analysis of variance by ranks with *P* value of 0.05 considered significant. Values are reported as mean ± SEM.

## Results

### Passive and active membrane properties

**Figure 1:**
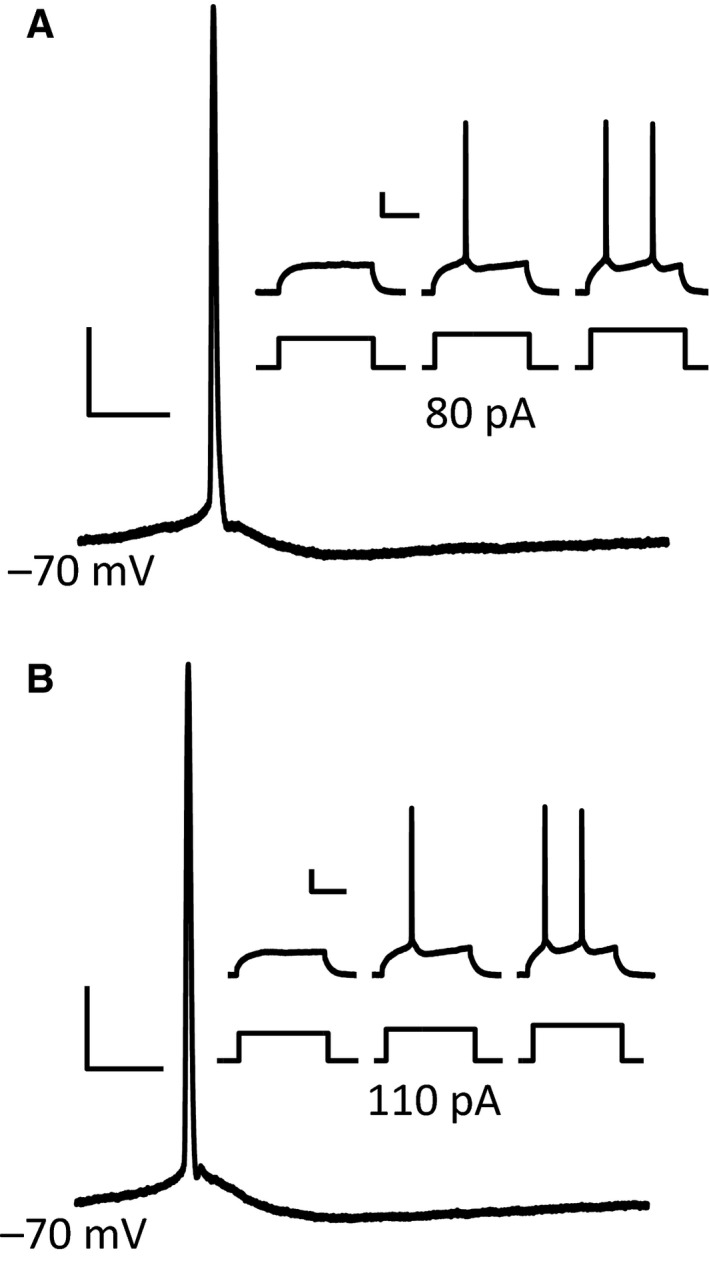
Action potential kinematics from layer 4 regular spiking neurons. Representative action potentials from a euploid (A) and trisomic (B) layer 4 neuron. The inset shows three consecutive 10 pA depolarizing steps for each cell with the rheobase value indicated under the middle trace. Although rheobase values tended to be higher in trisomic neurons the differences were not significant. The vertical scale bar indicates 20 mV while the horizontal scale bar indicates 20 and 100 ms for the main panel and inset respectively.

Neuronal network activity depends on the intrinsic properties of its constituent neurons. The intrinsic properties of hippocampal neurons in mouse models of DS are known to be altered with putative adverse effects on information processing in this structure (reviewed in Cramer et al. 2010). Therefore, we examined whether intrinsic properties of regular spiking (RS) neurons in the somatosensory cortex of trisomic Ts65Dn mice were altered when compared with their littermates. Layer 4 RS neurons from trisomic mice had a significantly increased membrane capacitance compared to euploid controls (94 ± 5 pF, 29 neurons vs. 70 ± 4 pF, 20 neurons respectively, *P* = 0.003). In addition, the specific membrane resistance was significantly reduces in RS neurons from trisomic mice compared to controls (2.6 ± 0.3 MΩ/pF vs. 4.6 ± 0.7 MΩ/pF, *P* = 0.007). These results, suggestive of larger neurons with leakier membranes, is consistent with changes observed in cerebellar granule cells (Usowicz and Garden 2012) and would be consistent with increased shunting in Ts65Dn neurons. In contrast, we did not find a significant difference in the resting membrane potential of RS neurons between the two genotypes (Ts65Dn: −64.8 ± 0.9, Euploid: −66 ± 1 mV; *P* = 0.47). Figure 1 shows a representative action potential from a euploid and trisomic layer 4 neuron and highlights the lack of significant differences between the two ploidies. In general, there were no statistically significant differences in the active membrane properties (Table 1).

**Table 1:** Active intrinsic membrane properties are unaffected in Ts65Dn layer 4 neurons

	Action potential properties							
	Rheobase (pA)	Amplitude (mV)	AHP (mV)	FWHM (ms)	Max rise (V/s)	Max decay (V/s)	Threshold (mV)	SFA ratio
Euploid	55 ± 8	112 ± 2	−12.0 ± 0.5	1.32 ± 0.04	231 ± 17	−113 ± 4	−40.6 ± 0.7	1.27 ± 0.06
Trisomy	73 ± 8	115 ± 2	−11.7 ± 0.9	1.27 ± 0.03	237 ± 12	−122 ± 4	−38.9 ± 0.8	1.29 ± 0.05
*P*‐Value	0.1	0.37	0.72	0.34	0.76	0.12	0.13	0.75

Recordings were performed in voltage‐clamp configuration. Statistical analysis was performed using a Student's *t*‐test (*P *< 0.05).

### Spontaneous synaptic activity is less frequent in trisomic mice

**Figure 2:**
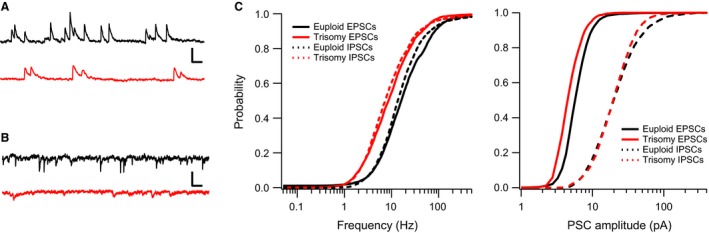
Spontaneous synaptic activity is decreased in layer 4 of the Ts65Dn somatosensory cortex. Representative voltage‐clamp recordings from euploid (Black) and trisomic (Red) neurons where IPSCs (A) and EPSCs (B) were isolated by holding the neurons at the reversal potential for the opposing currents. (C) Group data (EPSCs: 13 euploid and 11 triosmy cells; IPSCs: 11 cells for both euploid and trisomy) indicate a leftward shift in the probability distribution functions for both types of synaptic activity in trisomic mice. The vertical scale bar in represents 25 pA in (A) and 10 pA in (B) while the horizontal scale bar indicates 50 ms in both panels.

In the hippocampus of trisomic Ts65Dn mice, we found that spontaneous inhibitory synaptic inputs to CA1 hippocampal pyramidal neurons are significantly increased in frequency (Chakrabarti et al. 2010), a phenomenon that appears to contribute to aberrant synaptic plasticity in this structure. Unexpectedly, analysis of spontaneous electrical activity of layer 4 excitatory neurons in thalamocortical slices from Ts65Dn mice revealed an opposite trend. Here we find that the frequency of both excitatory and inhibitory spontaneous synaptic currents (E/IPSC) is significantly reduced compared to those in euploid littermates. This reduction in frequency is depicted in Figures 2A and B, which show representative recordings of IPSCs and EPSCs respectively from layer 4 neurons of euploid and Ts65Dn mice. Group data from all neurons (EPSCs: 11 neurons from 7 euploid mice and 11 neurons from 5 Ts65Dn mice; IPSCs: 13 neurons from 7 euploid mice and 11 neurons from Ts65Dn mice) are shown in the probability distribution vs frequency plots in Figure 2C. Both IPSC and EPSC curves for trisomic mice are significantly shifted leftward (toward lower frequencies) with 50% probabilities for IPSCs: 7.4 versus 13.7 Hz and EPSCs 8.7 and 16.2 Hz for Ts65Dn and euploid neurons respectively. The magnitude of the leftward shift is similar for EPSCs and IPSCs suggesting the balance between these opposing currents may remain relatively unchanged.

### Perturbations in spontaneous and evoked oscillatory activity

**Figure 3:**
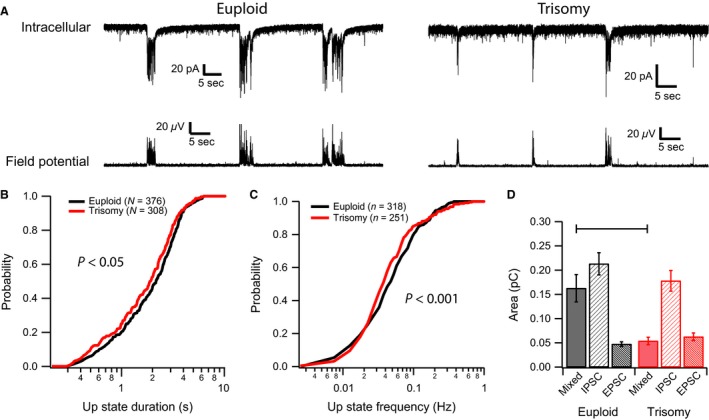
Spontaneous Up States are altered in Ts65Dn neocortex. (A) Representative intracellular and field potential recordings from euploid (Left traces) and Ts65Dn (Right traces) neocortex. The duration of spontaneously occurring Up States was significantly shorter in recordings from Ts65Dn mice (B) while the frequency of Up State occurrence (C) was slightly but significantly faster. (D) The Up State areas were similar between the two genotypes with the exception of those recorded close to the resting membrane potential (mixed, −70 mV holding potential, *P *< 0.001 Two Way ANOVA (analysis of variance) with Holm–Sidak post hoc comparisons).

The changes observed in spontaneous synaptic activity and passive membrane properties suggest that persistent network oscillations (Up and Down states), which depend on balanced excitation and inhibition, may be altered in the trisomic somatosensory cortex. We examined the kinetics of Up states in thalamocortical slices from euploid and Ts65Dn mice using a combination of intracellular and extracellular techniques. Representative examples of Up States from layer 4 neurons from euploid and trisomic thalamocortical slices are shown in Figure 3A. The distribution of Up State durations and frequencies (Figures 3B and C) from trisomic mice were shifted leftwards compared to euploid indicating that these network events are shorter (trisomy vs. euploid: 50% probabilities of 1.9 and 2.2 s, *P* < 0.05, KS test) and occur less frequently (trisomy vs. euploid: 50% probabilities of 0.038 and 0.048 Hz, KS test *P* < 0.001) in trisomic mice.

We also examined the charge transfer during the Up States by measuring the area of each event. Up States recorded in voltage‐clamp at −70 mV and containing a mixture of contributions from excitatory and inhibitory synaptic currents had a significantly smaller charge transfer in neurons from trisomic mice (solid bars in Figure 3D: 0.054 ± 0.008 pC vs. 0.16 ± 0.03 pC, *P* = 0.0008; *n* = 77 and 30 events for trisomy and euploid respectively). The decreased charge transfer in trisomic mice could result from either reduced inhibitory or excitatory drive to these neurons or by shifts in the intrinsic properties of the neurons themselves. To help isolate whether alterations in excitatory and inhibitory inputs contribute to this difference, a subset of recordings were performed with a Cs‐Gluconate based intracellular solution that enables isolation of each of these components (see Materials and Methods). Under these recording conditions, we found no significant difference in the magnitude of charge transfer for either inhibitory or excitatory post‐synaptic currents (hashed bars in Figure 3D). The inhibitory component had an average charge transfer of 0.18 ± 0.02 pC in recordings from trisomic neurons versus 0.21 ± 0.02 pC in those from euploid neurons (*P* = 0.26, *n* = 69 and 79 Up States respectively). The excitatory component had an average charge transfer of 0.062 ± 0.008 pC in recordings from trisomic neurons versus 0.047 ± 0.005 pC in euploid neurons (*P* = 0.1, *n* = 68 and 72 Up States respectively). As cesium blocks potassium channels, this result suggests that the changes in charge transfer seen during K‐based recordings result from changes in the intrinsic properties of the recorded neurons, such as their lower‐specific membrane resistance, and is consistent with increased shunting in Ts65Dn mice.

**Figure 4:**
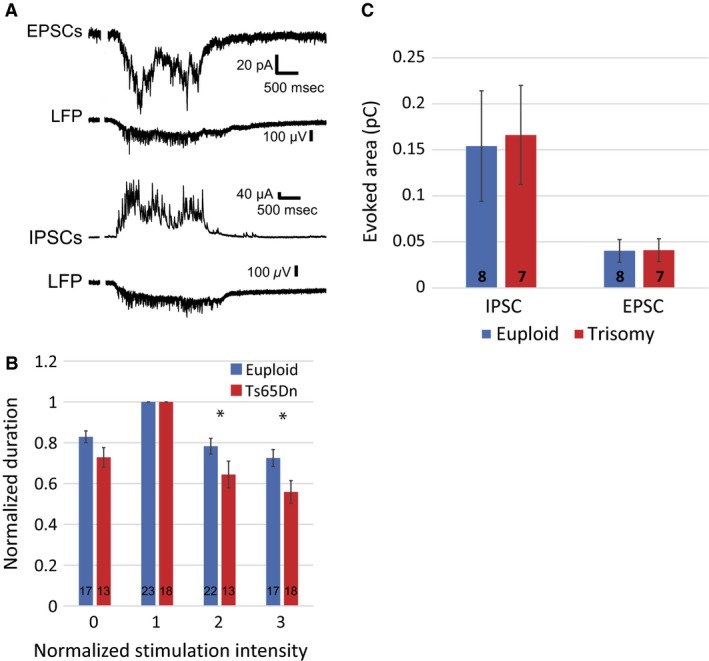
Deficits in Up States evoked by stimulation of the ventrobasal nucleus of the thalamus. (A) Example of excitatory inputs (top traces) and inhibitory inputs (bottom traces) in a single cell during Up States. (B) Evoked Up State durations declined as a function of stimulus intensity when normalized to the peak response values. This effect was significantly more pronounced in recordings from trisomic mice. (C) Although the evoked Up States tended to be shorter in trisomic slices there was no significant difference between excitatory and inhibitory charge transfer when each component was isolated.

The presence of intact thalamocortical connections in this slice preparation enables simulation of sensory inputs to the sensory cortex by stimulation of the ventrobasal nucleus of the thalamus. These stimulation evoked Up states tend to mimic those occurring spontaneously. A representative example of a stimulus evoked Up state is shown in Figure 4A. In this example, recordings were obtained with the Cs‐gluconate based intracellular solution that made isolation of EPSCs (top) and IPSCs (bottom) possible. We found that increasing the stimulus intensity beyond the threshold for evoking the maximal response tended to suppress the duration of the Up State (Figure 4B). This suppression was significantly greater in slices from Ts65Dn mice where at two and three times the most effective stimulation intensity the evoked Up State durations were reduced by 18 and 23% respectively. However, as observed for spontaneous events, the magnitudes of excitatory and inhibitory inputs were unchanged between genotypes (Figure 4C). The shortened duration of Up states at supra‐maximal stimulation intensities in trisomic slices may reflect enhanced feed‐forward inhibition in this condition. Indeed, an excessive inhibitory tone is a hallmark of hippocampal circuitry in the Ts65Dn mouse (Costa and Grybko 2005; Cramer et al. 2010; Best et al. 2012; Kleschevnikov et al. 2012).

**Figure 5:**
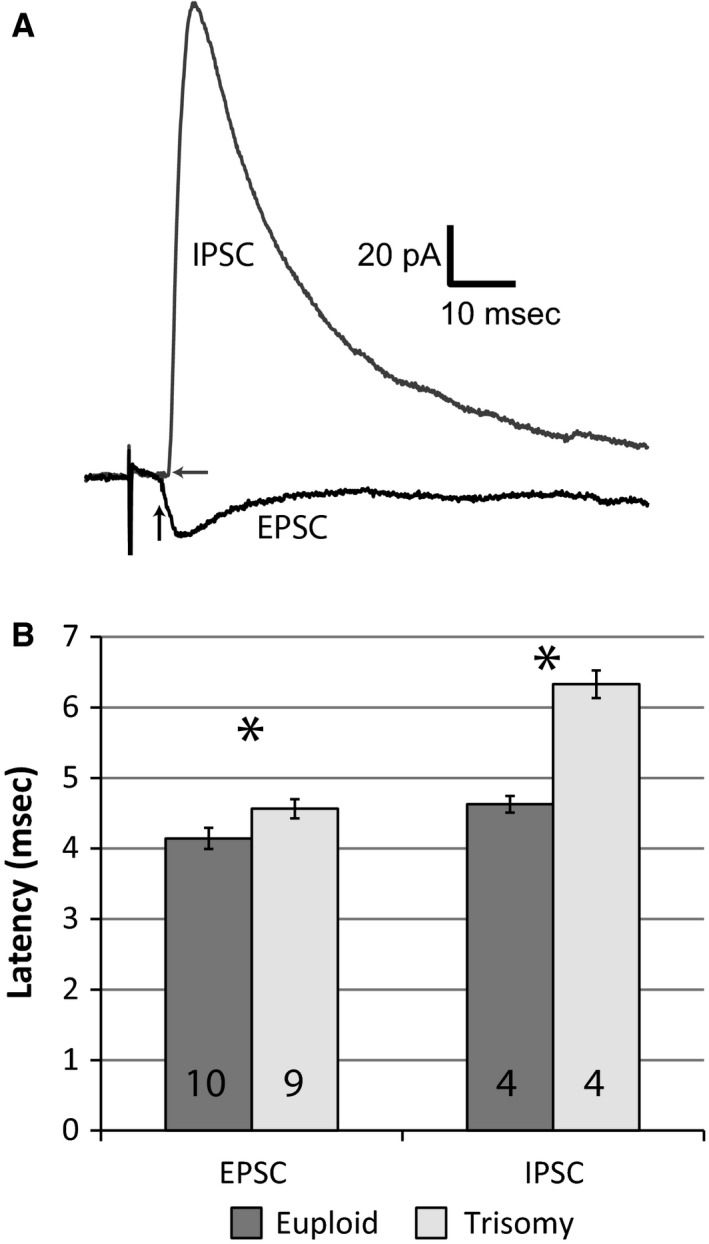
Delayed monosynaptic excitation and feed‐forward inhibition in Ts65Dn layer 4 neurons. (A) Representative example of a monosynaptic EPSC and polysynaptic IPSC in a euploid layer 4 regular spiking neuron. Arrows indicate the time of PSC onset. (B) Group data for EPSCs (*n *= 10 and 9 cells for euploid and trisomy respectively) and IPSCs (Euploid *n *= 4, Trisomy *n *= 4) reveal significant delays in both signals in trisomic networks. *P < 0.05, Student's *T*‐test.

Examination of the monosynaptic, short latency responses in layer 4 neurons arising from stimulation of the ventrobasal nucleus of the thalamus also revealed significant differences between genotypes (Figure 5). Both monosynaptic EPSCs and polysynaptic IPSCs arrived with longer latencies in recordings from Ts65Dn mice compared to those from euploid (Figure 5B). Monosynaptic EPSCs had a latency of 4.1 ± 0.2 ms (*n* = 10) in euploid neurons while the latency in Ts65Dn neurons was 4.6 ± 0.1 ms (*n* = 9, *P* = 0.02). Similarly, the polysynaptic IPSCs arrived with a latency of 4.6 ± 0.1 ms (*n* = 4) in euploid neurons and 6.3 ± 0.2 ms in Ts65Dn neurons (*n* = 4, *P* = 0.0003). The conduction velocity deficit apparent in the monosynaptic inputs from VBN to layer 4 stellate neurons would be consistent with impairments in oligodendrocyte‐mediated insulation which may also contribute to reduced excitability through shunting. Oligodendrocyte deficits may also affect glia mediated buffering further affecting network activity. The significant delay in feed‐forward inhibition in these neurons may be also result from of reduced intrinsic excitability of Ts65Dn layer 4 neurons as reported above. Together such changes would be expected to have a greater impact on the latency of polysynaptic inputs (IPSCs) as opposed to monosynaptic (EPSCs).

## Discussion

Cognitive disability is a uniform phenotype of Down syndrome. Although present with varying degrees of severity, impaired cognition in DS individuals negatively affects their independence and quality of life. A large body of evidence has been generated on hippocampal deficits that contribute to aberrant cognition in DS. These include morphological deficits at a cellular and network level beginning in utero (Sylvester 1983; Contestabile et al. 2007; Guidi et al. 2008) with functional deficits in spatial cognition through development (Pennington et al. 2003; Edgin et al. 2010).

While clearly important for cognitive processes, the hippocampus is not the sole structure involved in learning and memory. Interactions between the hippocampus and medial prefrontal cortex result in a gradual consolidation of memories within the neocortex (Takehara‐Nishiuchi and McNaughton 2008; Takashima et al. 2009) such that the consolidated memories may become hippocampus independent (Takashima et al. 2009; Nieuwenhuis et al. 2012). However, reactivation of consolidated memories can also involve, and once again become dependent upon, the hippocampus (Nader and Hardt 2009; Winocur et al. 2010). In addition to memory consolidation, regions of the neocortex, such as the prefrontal and parietal cortex, are also involved in working memory processes where changes in neuronal firing rates are observed during behavioral tasks requiring the temporary retention of information (Chafee and Goldman‐Rakic 1998; Sawaguchi and Yamane 1999; Pesaran et al. 2002). It is becoming increasingly evident that primary sensory regions of the neocortex also contribute to higher level cognitive processes (Postle 2006; Bancroft et al. 2014). Thus the deficits in processing sensory information observed in DS individuals (Babiloni et al. 2009; Bruni et al. 2010; Wuang and Su 2011) as well as DS mouse models (Martínez‐Cué et al. 1999; Scott‐McKean et al. 2010; Kuhn et al. 2012) may impact not only the regional modality but cognitive abilities in general**.** In spite of these reports, assessment of electrophysiological neuronal and network function within the somatosensory neocortex has not been performed yet. Therefore, using the Ts65Dn mouse model of DS, we sought to understand how the functional properties of neocortical somatosensory networks are altered by trisomy at the cellular and network level.

Passive membrane properties of regular spiking layer 4 neurons were affected by trisomy. Layer 4 somatosensory neurons from Ts65Dn mice had significantly greater membrane capacitances and lower‐specific membrane resistances. Cerebellar granule cells show a similar change in capacitance although the specific resistance of these neurons is greater than those from euploid mice (Usowicz and Garden 2012). However, in the hippocampus, the membrane capacitance of trisomic CA1 pyramidal neurons is smaller than euploid (Best et al. 2012). As cell membrane capacitance is proportional to the size of the neuron, these observations suggest that alterations in gene dosage present in Ts65Dn can have differential effects on the size of neurons across the brain. Indeed, direct observations of smaller pyramidal neurons in the Ts65Dn mouse (Dierssen et al. 2003) and reduced dendritic spines in neocortical neurons in DS humans (Suetsugu and Mehraein 1980) further highlight the complex interaction between gene dosage and the physical parameters of neurons. In the context of our investigations of network activity, it is likely that the increased capacitance and decreased‐specific membrane resistance in trisomic layer 4 neurons would have a dampening effect on network activity. Any incoming synaptic activity would produce a smaller change in membrane potential in trisomic versus euploid neurons. Thus somatosensory cortex networks would be expected to be more resistant to activation and less prone to sustain its activity compared to control. Our observations of a reduced frequency and duration of Up states in neocortical slices from Ts65Dn mice are in line with these expectations.

The overexpression of GIRK2 in the Ts65Dn brain (Harashima et al. 2006b) has been linked to hyperpolarization of the neuronal resting membrane potential and has been demonstrated for CA1 pyramidal neurons of the hippocampus (Best et al. 2007, 2012; Cramer et al. 2010). Despite overexpression of GIRK2 in the somatosensory cortex, the membrane potential of layer 4 neurons from this region were not significantly different in Ts65Dn mice. This suggests a potential homeostatic mechanism in this region of the brain capable of overcoming the GIRK2 induced bias or that GIRK2 overexpression is restricted to certain classes of neurons such as the pyramidal cells. Indeed, granule cells of the Ts65Dn cerebellum also show no change in resting potential (Usowicz and Garden 2012). Interestingly, unipolar brush cells of the Ts65Dn cerebellum express greater levels of GIRK2 than granule cells (Harashima et al. 2006a) further suggesting that a differential level of GIRK2 expression between cells within a given brain region can contribute to heterogeneity in membrane properties.

Although not significant, layer 4 neurons from the somatosensory cortex of Ts65Dn mice tended to have higher action potential thresholds and rheobases than those from their euploid controls. The shifts in these values likely arise from the altered membrane resistances of the trisomic neurons and would be expected to leave these cells less excitable when compared to controls. If similar changes occur in other cells of the neocortex then the entire neocortical network may be less excitable and could explain the reduced spontaneous synaptic activity we observed in these cells. These results could also explain the reduction in spontaneous network oscillations or Up states we observed in trisomic thalamocortical slices.

With respect to network activity, we found that layer 4 RS neurons of the somatosensory cortex receive less frequent spontaneous synaptic input in Ts65Dn mice when compared with euploid neurons. Both IPSC and EPSC rates were reduced (Fig. 2). The magnitude of this reduction was similar suggesting a potential attempt by the trisomic neocortex to maintain a normal balance between excitatory and inhibitory drives. This reduction is in contrast with findings in the hippocampus where the frequency of spontaneous inhibitory synaptic inputs to CA1 pyramidal cells is significantly increased, an effect that can be rescued by normalization of Olig1/2 expression levels (Chakrabarti et al. 2010). The diminished spontaneous synaptic activity is consistent with the alterations we observed in the intrinsic properties of layer 4 neurons. These changes in reduced‐specific membrane resistance and elevated capacitance would make the neurons more difficult to activate in trisomic mice leading to diminished overall network activity. Although not examined here, changes in the density of synaptic inputs could also explain the reduced frequency of synaptic events. Future experiments including recordings of miniature E/IPSCs will help determine if such changes are present in the Ts65Dn neocortex.

Changes in the components of neural networks would be expected to alter its functional properties. We assessed the magnitude of the impact changes in the functional properties of neocortical neurons have on network kinetics by examining the phenomena of Up states in Ts65Dn trisomic and euploid mice. These oscillations are generated by mechanisms intrinsic to the neocortex and rely upon balanced excitation and inhibition (Sanchez‐Vives and McCormick 2000; Haider et al. 2006). They are observed in vivo during slow wave sleep and some forms of anesthesia and may contribute to state changes in neuronal responsiveness (Destexhe et al. 1999; Steriade et al. 2001; McCormick 2005). These enhanced levels of responsiveness may assemble functional networks of neurons in a behaviorally relevant manner (Haider and McCormick 2009) providing a potential mechanism for behaviors such as working memory (Pesaran et al. 2002; Fries 2009; Mizuhara and Yamaguchi 2011; Palva et al. 2011) and sensorimotor integration (Murthy and Fetz 1992; Caplan et al. 2003; Cruikshank et al. 2012; Kilavik et al. 2012) These oscillations are also present in vitro in preparations such as the thalamocortical slice (Sanchez‐Vives and McCormick 2000) where alterations in Up state kinetics have been observed in a mouse model of Fragile X Syndrome (Gibson et al. 2008). We found that spontaneous Up states occurred less frequently and were of shorter duration while evoked Up states tended to terminate earlier in thalamocortical slices from Ts65Dn mice (Figures 3 and 4) These findings are again consistent with our observation of diminished intrinsic excitability of layer 4 neurons reduced spontaneous synaptic activity that could make the network both harder to activate and more difficult to maintain in this activated state.

Although not dependent upon external inputs for initiation or maintenance of oscillatory activity (Sanchez‐Vives and McCormick 2000), thalamic inputs to the sensory cortex are able to initiate Up state transitions (Rigas and Castro‐Alamancos 2007). We found that, addition to Up states stimulation of the ventrobasal nucleus of the thalamus evoked both monosynaptic EPSCs and polysynaptic IPSCs in layer 4 neurons of both ploidies but that the latency of both components were significantly delayed in trisomic slices. Deficits in myelination, potentially related to the overexpression of Olig1/2 (Chakrabarti et al. 2010), could explain the differences in latencies. Aberrant myelination in trisomic axons would be expected to slow conduction velocities. Since the mechanisms for Up state maintenance are intrinsic to the cortex, it is unlikely that differences in thalamic inputs contribute to the differences in Up state kinetics between the two ploidies. However, if aberrant myelination is a uniform feature throughout the trisomic brain, the correspondingly slower conduction velocities and higher transmission failure rates would be consistent with our findings of shorter and less frequent Up states.

In general, behaviorally, the difficulty in initiating and maintaining a type of network activity unraveled here could contribute to the cognitive dysfunction reported in these mice and present in DS individuals. It is interesting that, despite deficits in maintaining euploid levels of network activity, the balance between selectively determined excitatory and inhibitory drive (in shunting independent experimental conditions) appears similar between the two genotypes (Figures 3D and 4C) and suggests that the shunting deficit is not effectively compensated in Ts65Dn neocortex to maintain a proper level of network activity.

We find that the excitability regular spiking neurons in layer 4 of the somatosensory cortex are altered in the Ts65Dn mice and that spontaneous synaptic inputs to these cells, both excitatory and inhibitory, are decreased in frequency. Consistent with these observations, bursts of coherent network activity (Up states) were shorter and less frequent in thalamocortical slices from trisomic mice. Together these data suggest that the networks responsible for generating transitions between Up and Down states are perturbed in the Ts65Dn neocortex. Given that neocortical network oscillations are proposed to impact complex learning paradigms, and working or episodic memory, these alterations may contribute to the cognitive deficits associated with Down syndrome.

## Conflict of Interest

The authors have no conflicts of interests.
